# Applying systems approaches to stakeholder and community engagement and knowledge mobilisation in youth mental health system modelling

**DOI:** 10.1186/s13033-022-00530-1

**Published:** 2022-04-25

**Authors:** Louise Freebairn, Yun Ju Christine Song, Jo-An Occhipinti, Samantha Huntley, Pat Dudgeon, Julie Robotham, Grace Yeeun Lee, Samuel Hockey, Geoff Gallop, Ian B. Hickie

**Affiliations:** 1 Brain and Mind Centre, Faculty of Medicine and Health, University of Sydney, 94 Mallett Street, Camperdown, Sydney, NSW 2050 Australia; 2 Research School of Public Health, Australian National University, Canberra, Australia; 3 Computer Simulation & Advanced Research Technologies (CSART), Sydney, Australia; 4 School of Indigenous Studies, University of Western Australia, Perth, Australia

**Keywords:** Stakeholder engagement, Knowledge mobilization, Participatory action research, Community, Youth mental health

## Abstract

**Background:**

There is a significant push to change the trajectory of youth mental ill-health and suicide globally. Ensuring that young people have access to services that meet their individual needs and are easily accessible is a priority. Genuine stakeholder engagement in mental health system design is critical to ensure that system strengthening is likely to be successful within these complex environments. There is limited literature describing engagement processes undertaken by research teams in mental health program implementation and planning. This protocol describes the methods that will be used to engage local communities using systems science methods to mobilize knowledge and action to strengthen youth mental health services.

**Methods:**

Using participatory action research principles, the research team will actively engage with local communities to ensure genuine user-led participatory systems modelling processes and enhance knowledge mobilisation within research sites. Ensuring that culturally diverse and Aboriginal and Torres Strait Islander community voices are included will support this process. A rigorous site selection process will be undertaken to ensure that the community is committed and has capacity to actively engage in the research activities. Stakeholder engagement commences from the site selection process with the aim to build trust between researchers and key stakeholders. The research team will establish a variety of engagement resources and make opportunities available to each site depending on their local context, needs and audiences they wish to target during the process.

**Discussion:**

This protocol describes the inclusive community engagement and knowledge mobilization process for the *Right care, first time, where you live* research Program. This Program will use an iterative and adaptive approach that considers the social, economic, and political context of each community and attempts to maximise research engagement. A theoretical framework for applying systems approaches to knowledge mobilization that is flexible will enable the implementation of a participatory action research approach. This protocol commits to a rigorous and genuine stakeholder engagement process that can be applied in mental health research implementation.

**Supplementary Information:**

The online version contains supplementary material available at 10.1186/s13033-022-00530-1.

## Background

Globally, the magnitude of the burden of mental illness and its associated adverse human, economic and social impacts has been well described [3, 4]. In Australia, mental illness is the largest single cause of disability, with as many as one in five people aged 16–85 years experiencing a mental illness in any one year [6]. While mental illness across the life-course requires attention, more than half of the mental illness experienced in adult life has its onset in childhood or adolescence [7]. This has important implications for social, family, educational and vocational trajectories and, for the longer term economic and social future of the Australian community. However, best approaches for achieving improved system design, system strengthening, and resource allocation to improve youth mental health outcomes are unclear.

The need to understand and respond effectively to regional variation in the characteristics and drivers of mental ill-health, including suicidal behavior, across Australia was a major factor in the establishment of 31 Primary Health Networks (PHNs) in 2015 [8]. These not-for-profit organisations have responsibilities to undertake needs analysis, planning, coordination and commissioning of primary health care services and supports across their designated region. Although PHNs do not directly deliver services, they are funded to commission local service providers to deliver initiatives, including mental health and suicide prevention programs, in accordance with local population needs, contexts and priorities, as well as fostering critical collaboration among local stakeholders in their regions.

### Genuine stakeholder engagement in mental health research

User-led research and underpinning participatory research methods can lead to improved stakeholder engagement and active involvement in priority setting, knowledge mobilization (KM) and dissemination [9]. Mental health programs are typically implemented in complex environments where services can be fragmented, agencies have competing health priorities, limited funding and infrastructure, and unexpected crises such as political instability, natural disasters, and pandemics can be disruptive [9]. Such complex environments pose greater challenges in the implementation of mental health research programs. Therefore, stakeholder engagement is increasingly recognized as vital to successful and sustainable program implementation [10–12]. Despite such importance, there has been a clear lack of literature and research detailing the processes of stakeholder engagement in national and global mental health initiatives. Genuine stakeholder engagement affords stakeholders a place at the table, including them in the decision-making process, allowing them to hear plans, have a voice and actively contribute [13]. If stakeholders are permitted to advise, but researchers retain the ultimate decision-making power, this will lead to superficial or tokenistic, rather than meaningful stakeholder engagement [14, 15]. Therefore, an intentional partnership between researchers and stakeholders are both important to enable successful implementation of mental health programs [14, 15]. In the context of this research Program, genuine engagement means having flexible implementation strategies (described later) to be adaptive to stakeholder needs and facilitate active participation of a range of people [2]. Such engagement with service-users and community members from the commencement of research and implementation planning, can promote the needs and feasibility of research program objectives [16].

### Knowledge mobilization through a participatory system modelling process

Knowledge mobilization (KM) refers to the activities and approaches used to create and share research-informed knowledge [17]. Many terms are used to describe this process (e.g., knowledge translation or research to practice/action) however, the term KM was chosen for this protocol as it acknowledges that the process is emergent, multi-directional, complex, highly relational and context dependent [18, 19].

The potential for systems thinking and systems science methods to complement and strengthen KM approaches for complex health issues is increasingly recognized [19–23]. Systems approaches, including participatory systems modelling can assist in elucidating the behavior of complex problems, such as youth mental ill-health, and inform efforts to address them [24–26]. Systems approaches recognize that these challenging health issues occur within complex systems that are dynamic, interdependent, and evolving which need to be better understood to make positive, impactful change [26–28]. Haynes and colleagues articulate a theoretical framework that combines systems approaches and KM archetypes (groupings of KM activities) and recognizes the complexity and unpredictability of working within systems, such as mental health systems [19].

The utilization of a participatory systems modelling approach and KM archetypes as a theoretical framework facilitates a disciplined approach to stakeholder engagement for large-scale national mental health implementation programs. KM can be deployed as a critical strategy to improve health policy and practice, whereby the application of systems thinking and participatory modelling can be utilized to improve KM [18]. It is important to note that participatory action research (PAR) approaches and specific adaptations of PAR principles have been developed to address Indigenous research contexts. However, it should also be recognized that large national mental health programs will generally be poorly suited to Aboriginal and Torres Strait Islander people. This recognises the effects of colonization and ongoing social exclusion and the need for Indigenous knowledge to be developed by Aboriginal and Torres Strait Islander communities and people themselves. Therefore, a distinctive Aboriginal PAR (APAR) approach has been proposed by Dudgeon and colleagues [29].

### Implementation that is adaptive, contextual, and reflexive

Our program of research recognizes an approach that will apply participatory systems modelling (capturing the broader social drivers of youth mental health issues), digital infrastructure, and service innovation to develop a scalable and sustainable blueprint for broader national and international applications [1, 2]. This also requires a PAR approach which is a well-established research methodology, typically adopted when there is a need for action to address an inequitable situation [30]. It is often described as a reflexive cycle requiring gathering and reflecting as a group, planning action and inquiry, acting, observing and recording and returning to reflect further [31]. Researchers should approach PAR with humility, openness to learning and respect for participants’ own perspectives and expertise relevant to KM [32]. Previously, our research has used participatory modelling as a KM tool in Australian policy settings, whereby participatory methods placed stakeholders at the center of the process [18]. The process enables co-production of knowledge, facilitates transparency and confidence in model outputs to inform policy and program decisions [18, 33].

Principles for working in a culturally responsive way with Aboriginal and Torres Strait Islander communities, to support mental health and wellbeing, have been articulated by leaders in this field [34]. Aboriginal and Torres Strait Islander communities and people experience psychological distress at higher rates than other Australians, and the suicide rate among Aboriginal and Torres Strait Islander children and youth is around four times higher [35]. Explanations for this disparity centre on intergenerational trauma and the continuing experience of colonizing practices and attitudes, which undermine protective factors in Aboriginal and Torres Strait Islander cultures and communities. Therefore, it is essential that engagement with Aboriginal and Torres Strait Islander people is empowering and respectful of their strengths, capacities, and leadership. PAR approaches mirror Aboriginal and Torres Strait Islander community capacity-building practice [29], and may be appropriate for working with Indigenous communities provided they are sufficiently flexible to accommodate fundamental differences in perspective; for example the Aboriginal and Torres Strait Islander concept of social and emotional wellbeing [36] encompasses a broader, more holistic perspective than the Western notion of mental health and mental illness. An Aboriginal PAR approach recognises cultural differences and the need to acknowledge and facilitate the development of specific Indigenous knowledges by Aboriginal and Torres Strait Islander communities and people. It also recognises that the service systems surrounding Aboriginal and Torres Strait Islander community groups are likely to be experienced differently in terms of historical and institutional racism, and/or they may be culturally inappropriate.

#### Aims and objectives

This methods protocol describes the site and stakeholder engagement process to be implemented in the *Right care, first time, where you live* Program (the Program; see Box 1). The Program will engage with youth mental health system stakeholders in multiple Australian sites to provide decision support tools that are contextualized for each local community [1]. The Program aims to embed shared decision-making from commencement to completion and to facilitate the inclusion of diverse voices, particularly the voices of those with lived experience, their carers and Aboriginal and Torres Strait Islander people. These important groups have traditionally been limited in power when decision-making authority is shared with other stakeholders such as hospital system leads, health network leadership groups and academic researchers [37]. To support this aim, our program of work will make every effort to align and adapt the Program with the site and stakeholder’s institutional norms and existing practices [38]. This protocol has contributions from key stakeholders, aiming to facilitate a shared understanding of the approach used, to engage with stakeholders and communities in the *Right care, first time, where you live* research Program.

Box 1: Study context—overview of the research program: *right care, first time, where you live* This Program will apply participatory systems modelling to mental health, recognising the broader social drivers contributing to poorer mental health outcomes. The developed model can be used to simulate a range of cross-sectoral strategies for supporting young people and their mental health. The participatory process and methodology applied will enable partnering with local communities to collectively improve the trajectory for young people. Principles of a genuine shared decision-making process will be applied to the systems modelling [1], participatory modelling process [2], evaluation process [5] and the economic analysis. Such applications can be regionally implemented and provide sustainable health system decision support tools, digital infrastructure and service innovation. The implementation will be applied in eight geographically diverse sites across Australia (2 sites per year). This Program will facilitate regions to effectively respond to the challenges ahead and establish a coordinated service ecosystem that supports young people to access the right level of care, delivered early (first time) in the course of illness, and for a sufficiently long-period, to ensure they thrive. A comprehensive evaluation of the: (i) Feasibility; (ii) Value; (iii) Change & Action (Impact), and; (iv) Sustainability of the Program will be conducted alongside the Program implementation and also incorporates a PAR approach [5].

## Methods

**Fig. 1:**
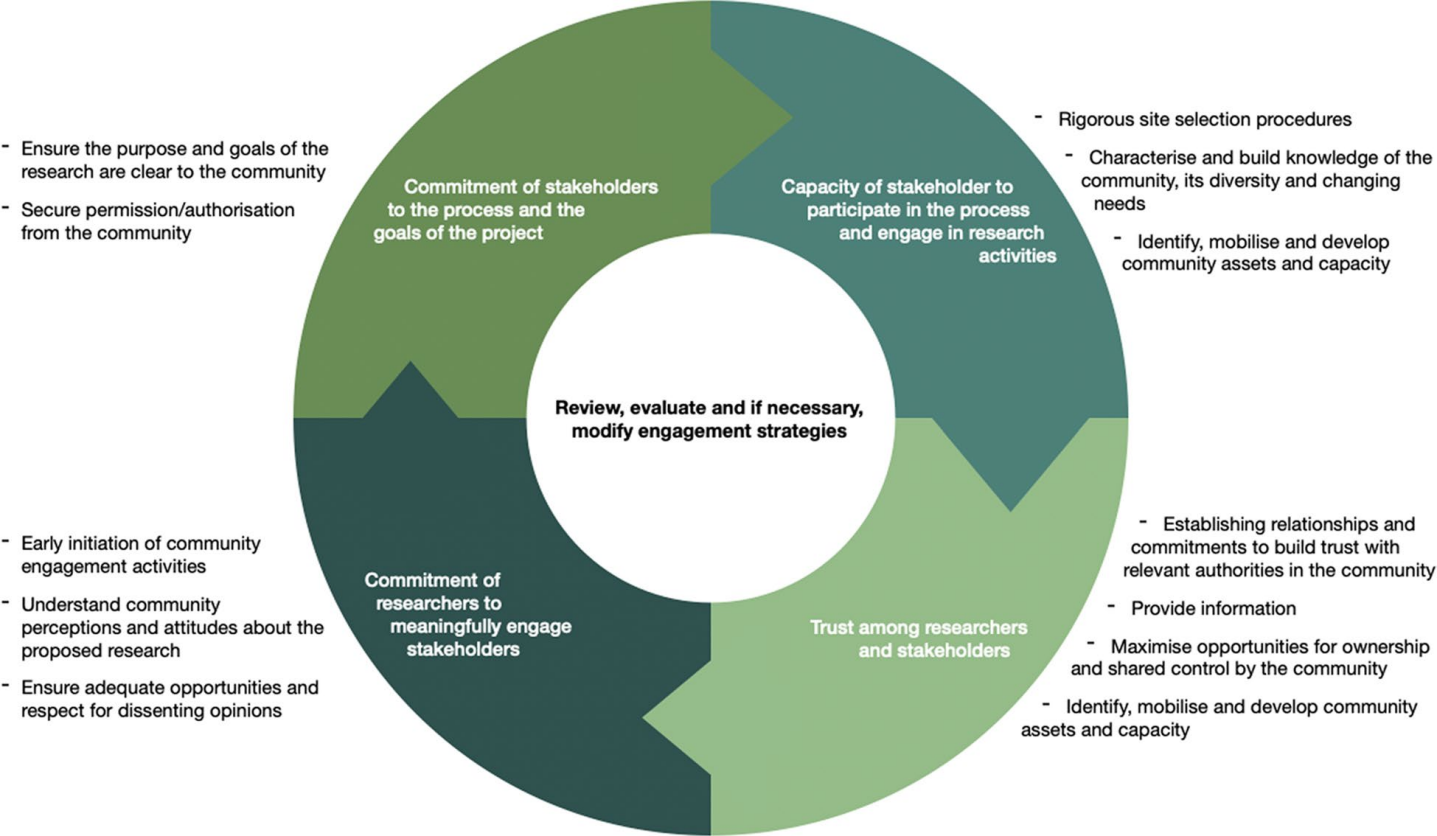
Proposed phases for stakeholder engagement [38, 40]

The importance of community engagement in research has been increasingly highlighted due to the concern of communities being harmed or exploited, particularly in developing countries and Aboriginal and Torres Strait Islander communities [39]. An effective community engagement framework has been proposed by Lavery and colleagues to articulate twelve points to consider and to avoid potential exploitation by researchers [6, 9, 40, 41]. We have adopted this engagement framework for our research Program (refer to Fig. 1) and established four phases to include: (1) site selection and capacity to participate; (2) establishing relationships and building trust; (3) committing to meaningful engagement (researchers); and (4) committing to the process (stakeholders).

### Phase 1: site selection and capacity to participate

A rigorous site selection process is vital in determining whether sites and their communities have the motivation and capacity to be active participants in community-based research [40]. The chosen sites will also operate across different states and territories and differences in state-based health service provision will be considered. The implementation plan for this Program will be phased across two sites each year in urban, outer-urban, regional and rural-remote regions. A total of eight sites will be selected to capture variation in:

- Key demographic factors such as socio-economic status (SES), education and employment status (focus on lower SES, education completion and employment);
- At least two sites (including urban/outer urban and regional/rural) specifically including a high proportion of persons who identify as Aboriginal and/or Torres Strait Islander and have significant health service organisations that are controlled and operated by the Aboriginal and/or Torres Strait Islander community
- Other demographic diversity factors (i.e., percent born overseas, culture, language at home other than English) that increase risk of mental ill-health or poor access to quality care; and,
- Diversity of existing health services infrastructure and community resources.

This approach will permit comparison of the factors that drive successful implementation of the systems modelling, monitoring, evaluation, and clinical service capacity building infrastructure in diverse settings. One of the most consistent findings from clinical and health services research is the failure of mobilizing research findings into practice and policy. Therefore, KM must be planned and implemented as an early and intentional approach [10].

It has been recommended that early initiation of engagement at the site selection process avoids pressing sites, when decision-makers are under pressure, to make rushed and ill-considered decisions purely to meet the timelines of researchers [40]. As a first step, expressions of interest will be sought from all potential sites that are interested in participating in the research Program. These sites would typically be the PHNs, that are responsible for regional suicide prevention service planning and commissioning. Sites will be provided a period of four weeks to express their interest in research participation. Following this, potential other sites will be invited based on which geographic locations needed to be filled remaining from the initial expressions of interest. A planning process is vital for implementation projects that seek engagement of multiple stakeholders to identify and address gaps that are needed to improve system effectiveness and efficiency [13]. This process will assist the identification of the appropriate administrative authority and relevant community stakeholders to ensure perspectives are sought through the early stages of the Program. This is likely to be especially important in the case of Aboriginal and Torres Strait Islander stakeholders and communities, where there may be a history of distrust of mainstream health authorities linked to experiences of discrimination [42]. Where these exist, local Aboriginal and Torres Strait Islander community-controlled organisations should always be invited.

The goals of the research in addition to other information necessary to the site will be actively shared using plain language, as meaning can be easily lost in the complexities of scientific jargon. Using accessible language will enable framing and justifying the relevance of research activities. Our previous health service research has also shown that the involvement of a local champion was a key facilitator of successful implementation [43]. Such local champions are not necessarily based on status but often earned and maintained by the individual’s competence, social accessibility, and conformity to the system’s norms [43]. The local champions will be influential in their health system’s communication structure and therefore, will likely have interpersonal and social networks that will engage with the knowledge, attitudes, and social norms of the target groups of the Program.

Following the initial meeting, a follow-up meeting with the site will be conducted to ensure that adequate information dissemination was provided by the research team to the site. As part of the rigorous site selection process, the meetings will enable the collection of specific information about the site to ensure sites are assessed for research implementation feasibility in a consistent and transparent way. An internal checklist by researchers will then be used to assess a site’s capacity in relation to the research Program goals and activities, including: co-design requirements and ethics approvals, governance frameworks, mental health needs and urgency, and cultural competency and Aboriginal and Torres Strait Islander governance (Table 1). This will then be followed by a feasibility meeting hosted by the research team to discuss the unique context and characteristics of the site that will enable feasibility of participation and also identify the barriers and facilitators of site participation. The sites are informed of the outcome of the site selection process.

**Table 1:** Site selection checklist questions

Themes	Example questions
Program activity	Is the site clear about the activities of the Program (particularly co-design requirements and ethics/governance approvals)?
Governance and stakeholders	Does the site have good governance and enjoy good relationships with other decision makers and stakeholders in the region?
	Has the site been involved in health service research studies?
Need and urgency	Does the site have a significant youth mental health and suicide burden and system challenges in addressing that burden?
	Does the site have a timeline to meet for internal decision-making?
Aboriginal and Torres Strait lslander peoples and communities	Does the site have demonstrably strong, collaborative relationships with local Aboriginal and Torres Strait Islander communities?
	Do relevant stakeholders or organisations in the site have a current Reconciliation Action Plan?

### Phase 2: establishing relationships and building trust

Establishing effective partnerships is key to successful implementation of participatory systems modelling projects. Early engagement with each site is important to ensure that the modelling will address their decision analysis needs. These discussions will help scope the systems model without changing the primary research question and overarching purpose of the work. Therefore, three site visits will be planned prior to implementation, to enable preparation to allow for a genuine and trust-building process between the researchers and the key stakeholders of the site (Table 2).

#### Site visit 1: leadership team (refer to Additional file 1: Appendix S1)

This is the first face to face meeting with the site and used to build rapport and trust with the site and their local champion. The research team prior to the visit ensures that attendees from the site are those that will be the key leadership stakeholders for the site, to ensure optimal implementation and championing of the Program. The scoping of the mental health needs, stakeholder networks, pressure points and political landscape will facilitate the identification of key decision-makers for the site and its region. The site will be asked to start early discussions with key stakeholders that will need to be present at Site Visit 2. Further, if there are key organisations that the research team could meet with at Site Visit 2, they can be identified at this visit.

The best methods of community engagement to advocate for the Program and engage with broader members of the community will be determined and identified by the site. The research team acknowledges that each site has its own specific mechanism of optimally engaging with the community, and therefore a specific community engagement plan that is adaptive and contextual will be developed collaboratively with the site. Funding will be allocated to all sites for community engagement purposes and to support a local community champion to work collaboratively with the research team. The research agreements that need to be reviewed will be discussed at this site visit to ensure there is ample time for review and execution prior to the implementation phase. This will also facilitate early and reciprocal understanding of research Program expectations to be explored and reviewed.

#### Site visit 2: operational team (refer to Additional file 2: Appendix S2)

This visit is critical to establish and discuss the mental health challenges for the local context and the priority mental health needs for the community. Site delegates will be encouraged to speak directly about some of the contextual issues and any other commissioning priorities that will impact or complement the Program. The stakeholder group that will be participating in the implementation process will be confirmed by the site and the proposed roles and expectations during the participatory process will be discussed. Further, the evaluation approach for the participatory modelling process will be discussed including how the recruitment approach will be established. The overall approach of the economic analysis will also be discussed, including the identification of key datasets.

#### Site visit 3: technical team (refer to Additional file 3: Appendix S3)

Key stakeholders that were identified in Site visit 2 will be invited to attend, including the data specialists from the site. The participatory systems modelling preparation will be discussed in detail, including model scope, data requirements, decision support priorities, interventions and outcomes that are likely to be prioritised by local stakeholders. Identified key stakeholders invited to the implementation process will be confirmed, including the identification of superusers of the systems modelling for the site. Superusers are local stakeholders with interest and capacity to be trained by the research team to interact with the systems model developed for the site and interpret findings. The research team’s systems modeller will present key data preparation requirements primarily by providing shell tables for completion and discuss any site-specific data needs. Data shell tables will be contextualised based on how the models will need to be stratified for each region.

**Table 2:** Site visit plan

Visits	Visit 1—(4 months prior to implementation)	Visit 2—(3 months prior to implementation)	Visit 3—(2 months prior to implementation)
Research team	Management team	Engagement team	Technical team
	Program leads, program manager	Systems modelling manager, economics lead, evaluation research manager	Program lead, systems modelling manager, systems modellers
Objectives	1. Rapport building	1. Rapport building	1. Rapport building
	2. Political landscape discussion	2. Participatory process preparation	2. Early discussion of model scope and decision support priorities
	3. Identification of key policy makers for site	3. Identification of key stakeholders that will be part of the model building process	3. Data preparation and discussion of shell tables
	4. Determining best methods for community engagement	4. Identification of super-users of systems modelling	4. Shell tables will need to be contextualised based on how models will be stratified per region
	5. Research collaboration agreement review	5. Evaluation framework preparation	
	6. Site visit plan discussed	6. Economic data preparation	

### Phase 3: committing to meaningful engagement (researchers)

This research Program aims to enable commitment by the research team to maintain sustainable and genuine partnerships with the sites and their communities. An advocacy plan for this research Program has been co-designed with lived experience participants and will be mapped against the objectives of the Program, focusing on awareness, engagement, and integration.

#### Community engagement strategy

A community engagement strategy will be deployed to support the research team’s commitment to meaningfully engage with the community and to initiate community engagement activities early. This will result in increased opportunities for KM in this Program [40]. The types of engagement opportunities will be discussed collaboratively [44] with each site and the research team working together to arrive at the optimal strategy that meets the site and their community’s needs. A variety of different approaches will be applied by the research team to build awareness, engagement, and integration with the community, based on local needs, desires, and resources available [45]. The collaborative consultation with sites is critical in discussing optimal dissemination methods through social media, podcasts, surveys, and consistent messaging to the local community [46, 47].

Social media offers significant opportunities to expand the reach and engagement of health messages [48]. Social media platforms including Twitter, Instagram and YouTube will be used to engage with each sites’ local community. The use of tagging and hashtags will also be deployed to allow the research team to engage not only with the local community at each site, but also to disseminate to a wider community nationally and internationally. Increasingly, podcasts have been considered a successful mechanism to disseminate information, particularly research information, and normalise academic experiences [49]. We have partnered with a youth lived experience podcast to capture the journey each site will undertake through the implementation phase of the research and disseminate this to the community. The podcast programming will focus on speaking with and learning alongside expert participants and young people from each site to embed and document their voice and community knowledge.

A brand is a valuable asset as it provides a means for consumers to recognise and specify a particular offering, should they wish to choose it or recommend it to others [50]. Program resources have been developed for each site to utilise and ensure that the awareness of the objectives and aims of the Program are delivered in a consistent manner to the public, community and relevant regional, state and national agencies. These include:

- Program logo to depict the participation of many different community members for one common goal;
- Infographics to highlight the messaging in the logo and bring a consistent colour pallet and feel to the Program;
- Videography to capture each community story (a ‘video diary’) which will continue to be developed across the lifecycle of the Program and will be used to engage communities, record workshop progress and explain the Program to the community in a succinct, informative and emotive way; and
- A Program information flyer will be developed as an engagement tool that sites can utilise to increase participation of expert stakeholders to the workshops through existing local alliances [51].

##### Community capacity building

Successful community engagement ensures the community benefits from the research (e.g., learning new skills or refining knowledge) [52]. The research Program will enable opportunities for increasing community and policy maker understanding, building capacity and KM. Superuser training is one of the measures to ensure sustainable continuation the Program beyond the implementation timeframe. This training will upskill nominated persons from within decision making and/or primary partner agencies who will be supported to build systems modelling knowledge in how to use and interpret findings from the systems model developed for their community. Following this interactive training the superusers will have competency in using the model to explore policy scenarios, and interpret and describe modelling findings in reports, policy briefs, business cases, and to support community advocacy even after the research Program is completed [13]. The research team will provide an ‘Insights Brief’ that will showcase the key findings and insights from the systems modelling process, including an explanation of the results. This will serve as the main communication output provided to sites and key stakeholders to support advocacy for changes or upscaling of service provision within their community.

The identification of local champions to support and translate the messages emerging from the Program in each local area will ensure the workshop findings can reach those not traditionally engaged in heath messaging and planning addressing health information inequality [43, 48]. The research team will fund and engage with a local champion to facilitate optimal engagement with the local decision makers, those who will benefit from service changes and improvements and community members who support others to navigate service structures. They will engage with their broader community to facilitate contributions to the modelling beyond the workshop participants and will disseminate findings that have emerged from the systems modelling workshops to the community. This wider engagement with the community will assist in building community confidence not only in the results but also give opportunity for the research team to hear from a diverse range of mental health voices across the community [53, 54].

#### Working with Aboriginal and Torres Strait Islander people

Where there is an Aboriginal and Torres Strait Islander population focus, this Program will seek to develop genuine partnerships and inclusion. For example, community members will be offered employment opportunities and included in governance processes for the site, while local community-controlled organisations will be invited to advise about appropriate people and organisations to participate in consultation and co-design processes. It is essential to recognise the central role of culture, community, and Country in social and emotional wellbeing, and how these ideas differ from Western concepts of mental health and illness [34]. Processes that support self-determination and cultural inclusion are also essential. The approaches to working with Aboriginal and Torres Strait Islander people and communities in this Program are informed by the literature and the author’s experiences implementing previous projects [29, 34].

1. Respect for Aboriginal and Torres Strait Islander governance:
  There is a strong commitment to building relationships of trust with Elders and community leaders and members that will sustain the Program and ensure the systems modelling addresses community priorities. This includes commitments: to be open and transparent about Program objectives, intentions and actions; to recognise the expertise that people share, including by compensating them appropriately; and to support community capacity building.
  It is important to work under the leadership and governance of Aboriginal and Torres Strait Islander communities and organisations, recognising that self-determination and decision-making autonomy are core foundations of effective suicide prevention for Aboriginal and Torres Strait Islander people.
  Local protocols will be observed, including ensuring all major meetings are opened by a community Elder and include a Welcome to Country, and meeting and consultation arrangements take account of cultural protocols as required.
2. Cultural safety:
  Working with local Elders and community members, the Program is committed to the full inclusion of Aboriginal and Torres Strait Islander people, valuing and supporting their human rights and cultural differences.
  Recognising that people’s culture and life experiences may require alternative consultation approaches, the Program will be open to flexible ways of meeting and decision-making, including yarning circles and spontaneous conversations at the request of the community. Time and space will always be made available for people to express their experiences and knowledges and what is most important to them [36].
  The loss of young people to suicide is common in Aboriginal and Torres Strait Islander communities [55]. In meetings where suicide is discussed, people may express grief and trauma, as well as anger about their experiences with health systems and institutions, and it is important to respectfully listen.
  The Program will support communities’ own understanding of mental health and/or distress, and not impose clinical or other external models.
  Consultations and communications will use strength-based language anchored in positive concepts of social and emotional wellbeing, avoiding academic and bureaucratic language that might exclude Aboriginal and Torres Strait Islander people.
  The Program recognises the importance of the role of culture in social and emotional wellbeing, including traditional healing practices and reconnection to Country and cultural practices [56].

#### Working with people with lived experience

Consumer and carer contributions and inclusion will be routine practice throughout the research activities involved in the Program [2]. People with lived experience of mental ill-health will be included both as stakeholders in the research and as members of the research team. Supporting an inclusive culture may involve allowing additional time for consumers and carers with the research team to clarify concepts and explain the systems model and engage with the user interface of the systems model that will be developed for each site. The practical strategies to support inclusion of people with lived experience of mental ill-health and their carers in this collaborative process include being conscious of language, providing a safe and supportive environment, facilitating access, and ensuring diversity in lived experience and cultural background. The procedures for working with people with lived experience in this study are informed by the literature [57–64], including the best practice principles for supporting consumer and carer participation in mental health research described by the National Mental Health Commission [63] and the authors’ experiences implementing previous projects in the mental health sector.

1. Language:
  Language use and word choice has a significant impact on all people. This is particularly applicable in individuals with lived/living experience of mental ill-health as a result of significant disadvantage, trauma, poverty, physical health quality, and stigma.
  It will be critical to ensure that choice in words and language used throughout the Program are inclusive and respectful and do not lead to further disadvantage and/or social exclusion.
  Language use should aim to be age-appropriate, respectful, non-judgemental, jargon free and accessible to any individual no matter their socio-economic and/or education background.
  The language should be person-centred i.e., ‘person with mental ill-health’ rather than ‘they are mentally ill’, and recovery oriented, conveying the potential for hope and opportunity.
  Communication should always be strengths focused rather than limitations focussed whilst being sincere. The Mental Health Coordinating Council provides an extensive and practical guide for using Recovery Oriented Language [65].
2. Safe and supportive environment:
  Safe engagement means that appropriate supports are available for anyone, not just people with lived experience of mental ill-health, to engage in the research.
  Practically, this may include being explicit about workshop guidelines/protocol for safe and acceptable disclosure, ensuring that participating individuals have access to and are aware of supports available (e.g., debriefing, referral to professional support, allowing people to take breaks when needed, observing levels of psychological distress within the group, checking in with participants, and offering support where appropriate).
  Workshop facilitators and support staff will ensure that large and small group discussions are respectful and inclusive for all participating individuals (e.g., valuing contributions from all participants, minimising instances where people are interrupted, cut off, or not listened too and minimising the use of jargon).
  Informal conversations during breaks, and outside of workshops, can be used to facilitate further engagement opportunities and encourage participation in the process by providing additional information, clarification, and context to the collaborative modelling process, method, and contribution opportunities outside of the formal workshop process (e.g., sharing stories and experiences of mental health system in direct one-on-one conversation with the modelling team member/s).
3. Facilitating access and ensuring diversity:
  The choice of location, venue, and timing for workshops will need to consider the needs of participating individuals (e.g., transportation, standard mealtimes, and any community-specific commitments).
  Barriers to participation can include distance and travel-times; environmental barriers also include individual requirements such as mobility access, physical space for ‘time-out’ breaks during workshops, dietary needs, and allowing sufficient time for participants to prepare for workshops (including organising of other commitments).
  Ensuring diversity means including and valuing contributions from people with varying cultural backgrounds (this includes their supportive others).

### Phase 4: committing to the process (stakeholders)

Collaborative and genuine partnerships are developed and sustained when the constituent members contribute their perspectives, resources, and skills creating an opportunity for research synergy, allowing the partnership to obtain outcomes that no one constituent member could have produced on their own [13]. To enable this in the context of this Program, engagement of sites must be conducted in a sustainable way to build partnerships that strengthen their communities. Implementation research shows there are four key constructs to consider when engaging multilevel stakeholders in the research process [38]:

i. Commitment of stakeholders to the process and the goals of the project;
ii. Capacity of stakeholder to participate in the process and engage in research activities;
iii. Commitment of researchers to meaningfully engage stakeholders; and
iv. Trust among researchers and stakeholders.

Although eight sites will be initiated at differing timepoints, the Program will aim to ensure all sites and the research team continue learning from each other throughout the timeline of the Program. There is a critical difference between going through the empty ritual of obtaining stakeholder feedback and genuinely providing stakeholders the real power needed to affect the research process and resulting outcomes [37]. Further, if these relationships are established early, greater trust can be built with communities [40]. The participating eight sites will be invited to annual symposiums to ensure that all sites are considered key participants throughout the Program. The symposiums will be an opportunity to share experiences and celebrate milestones; identify anticipated and unanticipated outcomes; incorporate key lessons; and consider optimization of Program delivery and progress.

### Analysis: theoretical approach to guide the participatory action research (PAR)

The theoretical model for applying systems approaches to KM processes described by Haynes et al. [19] will be adopted as a framework to inform stakeholder engagement strategies and KM methods for our research. The model outlines how systems thinking concepts, including leverage points to target, intersect with KM archetypes [17] and how they can be applied in practice to guide engagement strategies. Three KM archetypes have been identified as particularly relevant in the context of this Program of research, and they are: producing and disseminating knowledge; researching in practice; and fostering networks. For this research, an additional domain has been applied that focuses on working with Aboriginal and Torres Strait Islander communities.

The framework [19] outlines a hierarchy of leverage points for effecting change in complex systems ranging from elements, e.g., resources or practices, to structure and rules, to system paradigms [26, 66, 67]. Change is harder to achieve at higher levels of the hierarchy i.e., paradigm shift, however, when change is successful at this level it is more likely to be transformational [26]. When applied to stakeholder engagement and KM strategies for this research, each community engagement site is viewed as its own complex system and will be analysed to determine the change levers that are available and the strategies that may support effective KM and positive system change. The practical strategies for applying a systems approach to stakeholder engagement and mobilizing knowledge in the *Right care, first time, where you live* Program are described in Table 3.

**Table 3:** Practical strategies for applying a systems approach to mobilising knowledge as a theoretical framework in the *Right care, first time, where you live* Program

KM archetypes	Leverage points for changing complex systems					
	Paradigms	Goals	Structure and rules	Relationships and power	Feedback	Elements
Producing and disseminating knowledge	Apply a ‘systems lens’, using methods for analysing and responding to complexity	Be clear about the goals of collaborative knowledge production for the project including whether and how they fit with the goals of the local system –engage with local leaders who are in the strongest position to make this impactful	Establish a governance structure early to ensure a collaborative process from an early stage to ensure there are no surprise expectations	Reflection on whose knowledge is being privileged through the participatory modelling process and with what impacts. Aim to ensure that deliberative processes provide opportunity for a diverse range of knowledge to be incorporated into the model development	Provide clear and accessible preliminary findings to participants and incorporate their views in final papers and reports	Develop resources to facilitate learning, capacity development and co-production at each modelling site
	Complex problems are multifaceted: embrace interdisciplinary approaches	Engage with local stakeholders to understand their policy/practice challenges before trying to produce knowledge to influence change. Make the research useful for them	Include shared ethical approaches and governance standards to data sharing and research processes through collaborative agreements	Use implementation strategies to facilitate inclusion of people from a range of backgrounds, including people with lived experience, carers, Aboriginal and Torres Strait Islander people, people from culturally and linguistically diverse backgrounds and LGBTIQ + community	Discuss views that are at variance with model findings	Use creative strategies to communicate with and build systems thinking capacity among community partners and other stakeholders taking into consideration specific methods to engage with sub-groups for example, young people, Aboriginal and Torres Strait Islander communities
	Reflect on what knowledge is valued and most fit-for purpose	Look for opportunities to optimise benefits for communities through the exploration of ‘multi-solving’ approaches e.g., exploring portfolios of interventions that provide maximum positive impact	Provide knowledge on timelines and methodology proposed that is contextual to the stakeholders and the community		Resolve complex problems using iterative action, reflection and deliberation, sharing ideas and experiences as well as formal knowledge: build this into the participatory process	Learn about and from the context through PAR methods
	Question traditional hierarchies of evidence: do not automatically privilege a particular type of evidence if it is at the expense of “ecological fit” (local relevance and applicability)		Learn early the community and governance structures of stakeholders and their agencies to ensure knowledge production is accordingly adaptive and targeted		Engage stakeholders in interpreting and strategically communicating knowledge from the research, e.g., blend community stories with epidemiological data to educate the media and other stakeholders about human costs and contextual realities	
	Engage with local stakeholders and use participatory methods to build in relevance, applicability and knowledge translation. Help participants’ reflect on their worldviews and frames of reference by including stakeholders with a diverse range of knowledge and experience					
	Embed system science methods, e.g., systems modelling, to facilitate shared understanding of the challenges and improvement opportunities for local youth mental health systems					
Researching in practice	‘Diagnose’ the context to facilitate the selection of appropriate implementation strategies	Consider the system’s current and historical dynamics and how they impact on the future trajectory	Be flexible in implementation that is guided by principles and ‘form” rather than strict adherence to rules	Encourage local ownership of the research	Use knowledge to inform action through existing feedback (communication) loops and by establishing new feedback loops	Utilise methods that capture the views and experiences of key stakeholders
	Recognise that knowledge evolves and must be considered in context	Engage with local stakeholders to ensure that measures and outcomes reflect their interests and priorities	Focus on adaptation, interdependencies and being responsive to emerging and changing local needs	Work with local champions to communicate ideas, get feedback and adapt implementation strategies for a contextualised “best fit”	Use and invite stories to surface tacit knowledge	Work towards “best fit” practices that optimise reach in the community
	Recognise that this research is occurring within an existing complex system and should be empowering rather than prescriptive for local participants	Design shared goals	Foster local organisational and individual learning and development	Value local innovations that have been shown to work well for local communities	Use reflexive practices to learn, adapt and improve processes	Use learning oriented implementation
	Be cognisant that complex problems can be shifted but are rarely solved. Systems change takes time to embed and show results					Support local capability development
Fostering networks	Intentionally form networks across disciplines and stakeholder groups	Develop a clear vision for fostering networks within each community site	Be transparent about roles, structures and processes for decision making	Identify and work closely with local ‘connectors’ to facilitate relationships	Embed an open and reflective culture to facilitate learning across networks	Look for opportunities to facilitate full participation of local stakeholders in the research collaboration
	Aim to bring together diverse perspectives and develop a shared understanding of the local youth mental health system	Ensure that membership of the network is broad and inclusive	Articulate and, where possible, address barriers to knowledge sharing	Utilise methods that foster inclusivity, giving all participants a voice (eg., providing funding for each site to support community engagement)	Continuously review and adapt communication processes, KM strategies and goals to optimise local impact	Provide platforms for sharing knowledge
	Utilise system science methods, e.g., social network analysis, to map and analyse local networks and how they change over time		Consider where networks formed through this research can be embedded and sustained in the local system	Value the quality of relationships as highly as project delivery	Ensure that participants are kept well-informed about progress and have a say in decision making about project priorities	Embed evaluation strategies that capture the changes in social networks
	Use formal and informal networks and methods to communicate			Focus on building mutual trust and respect		
Working with Aboriginal and Torres Strait Islander communities	Recognise that the continuing process of colonisation forms the context of Aboriginal and Torres Strait Islander people’s lived experience of suicide, and that this requires a distinctive response to ongoing injustices	Ensure that knowledge and other benefits generated are genuinely accepted by and shared with Aboriginal and Torres Strait Islander communities	Be open to changing structures and processes in response to the advice of Aboriginal and Torres Strait Islander Elders and community leaders	Recognise the leadership of Aboriginal and Torres Strait Islander Elders and community leaders	Be prepared to accept feedback from Aboriginal and Torres Strait Islander people, in any form and at any time	Embed the Aboriginal and Torres Strait Islander concept of social and emotional wellbeing in all the work [77]
	Recognise the role of intergenerational trauma in Aboriginal and Torres Strait Islander suicide and be prepared to accept people’s grief, loss, distress or anger	Understand the genuine, reciprocal inclusion of Aboriginal and Torres Strait Islander people in this work as an act of reconciliation	Recognise the importance of engaging according to cultural protocols	Learn how to work under Aboriginal and Torres Strait Islander governance and leadership and respect the community’s own systems of authority when working with them	Make strong efforts to support Aboriginal and Torres Strait Islander leaders’ and communities’ participation in information sharing and iterative project development, recognising that they may have different engagement needs and preferences	Ensure all aspects of the Program conform to Aboriginal and Torres Strait Islander ethical principles [78]
	Apply Aboriginal and Torres Strait Islander knowledges and evaluation protocols wherever appropriate. In particular, acknowledge the holistic concept of social and emotional wellbeing as a foundation for preventing suicide		Support Aboriginal and Torres Strait Islander people’s governance and ownership of data that relates to them; collect and share data in a form that is valuable to Aboriginal and Torres Strait Islander leaders in pursuing their own community objectives	Work to develop strong reciprocal relationships of trust with Aboriginal and Torres Strait Islander leaders before attempting to transact business		

It is important to note that the Program will need to take account of the current policy directions, expenditure priorities and accountability mechanisms that have been put in place by both federal and state governments and consider how these impact on both the local system and the implementation of this Program. Their impact may be supportive of positive changes in the system or they may create barriers to progress. Regardless, there are likely to be “pressure points” that are contextual for each local site and will result in decisions and judgements needing to be made and compromises sought. In this context, it will be critical to be consistently alert to both the research team’s own values and biases and the values and biases of the stakeholders who participate in the Program [68].

As detailed in Program’s evaluation protocol [5], and to ensure a transfer of learnings and research quality assurance process, a PAR approach will be embedded to draw insights from the evaluation process through reflective research team discussions at key time points in the project e.g., following each site visit and participatory workshop. This process will seek to highlight the importance of having a disciplined but adaptive approach to site and stakeholder engagement when conducting research with health services. Internal team reflective discussions will be recorded for qualitative analysis opportunities and transfer of learnings. Based on the theoretical framework described above and the practical strategies outlined in Table 3, key questions will be discussed by the research team at key time points will include:

1. What strategies/actions are we putting in place to impact as many system leverage points as we can?
2. How are the system leverage points being implemented?
3. Do we need to change/modify strategies?
4. Are there gaps in what we are doing i.e., leverage opportunities that we are missing?
5. Are we ensuring the contributions of different stakeholder groups are balanced? e.g., young people with lived experience of mental ill-health.

## Discussion

Over 20 years in Australia, there have been five national mental health plans, and the urging of transparent strategies to implement accountabilities of such national plans, including the challenges arising from funding insecurity and resource allocation [69, 70]. Mental health services in Australia are plagued by service fragmentation and, while there is growing recognition of the importance of co-designing policies and services with people who will use them, this is limited in practice [71]. There are few examples of rigorous, co-designed implementation plans that are intended to address the inequities of distribution in the availability of mental health resources [71]. These services are incorporated into various, local human service systems and therefore, it is vital for multiple levels of stakeholders to be included in decision-making to enable system strengthening and research implementation design processes to inform it [72]. A greater understanding of different stakeholder perspectives may lead to improved collaboration to enhance health services and service delivery [72]. This protocol outlines our commitment to meaningful stakeholder engagement by using reflective KM and systems science approaches to support active participation in the research and enabling shared decision-making that respects diverse needs, perspectives, and interests.

The research Program will be implemented across various geographically and demographically diverse regions across Australia and includes regions with high populations of Aboriginal and Torres Strait Islander people. The process of stakeholder engagement will be iterative and flexible to accommodate the needs of the different communities and contexts [13] and support the engagement and inclusion of diverse stakeholder voices. Implementation of innovative research Programs like *Right care, first time, where you live* requires the consideration of multiple points of leverage and contexts that factor into the social, organisational, economic and political context of each community [73]. Therefore, input from various stakeholders that represent these different contexts are vital to address any implementation challenges that may require research Programs to be adaptive, to enable positive and sustained implementation [72]. This research draws on a theoretical framework for applying systems approaches to knowledge mobilisation that is flexible and can guide implementation approaches to accommodate the needs of participants [19] and the PAR approach. When engaging with communities with a high proportion of Aboriginal and Torres Strait Islander people, the approach will be guided by the specifically adapted APAR methodology previously published [29].

Stakeholder engagement initiated in the early stages of research can support the translation and interpretation of findings and is likely to accelerate the actionability of research outcomes [74, 75]. There is growing interest in and demand for research that focuses on stakeholder engagement, as it can facilitate the reorientation and improvement of research implementation, reduce uncertainties, accelerate the adoption of meaningful findings, and ultimately improves decision-making processes and health outcomes [76]. However, despite this growing interest there is limited evidence on the demonstrated value of research engagement with stakeholders and the KM such engagement could enable. Therefore, documentation and correspondence of clear objectives and shared vision of the research Program with stakeholders must take place early and throughout the research process. This also requires consistent communication with stakeholders to reconfirm priorities as efforts and interest on common goals can wane [9]. Initiating engagement early and taking the time to develop shared vision and reciprocity is vital to fostering trusting relationships with stakeholders [40]. The research protocol utilises a period of one year prior to implementation, to acknowledge early and sustained stakeholder engagement will facilitate the KM required to enable maximal research and community outcomes.

This protocol outlines an applied approach to engage youth mental health system stakeholders in a participatory systems modelling and digitally-enhanced care multi-site research Program. The Program aims to develop and provide enhanced system level decision support methods that are contextualised for diverse communities. The practical and theoretical approach to stakeholder engagement is described in this protocol to openly articulate the values and intended approach of the researchers in undertaking this research. The protocol also provides an example of how rigorous stakeholder engagement can be undertaken in mental health service research that can be used and further developed by researchers.

## Supplementary Information

**Additional file 1: Appendix S1. **Site visit 1 agenda.

**Additional file 2: Appendix S2. **Site visit 2 agenda.

**Additional file 3: Appendix S3. **Site visit 3 agenda.

## Data Availability

Not applicable.

## Abbreviations

BMC: Brain and mind centre
CALD: Culturally and linguistically diverse
KM: Knowledge mobilization
NGO: Non-government organisation
OECD: Organisation for Economic Co-operation and Development
PAR: Participatory action research
PHN: Primary health networks
SES: Socio-economic status
