# Supplementary material for: Applying systems approaches to stakeholder and community engagement and knowledge mobilisation in youth mental health system modelling
Source: Int J Ment Health Syst. 2022 Apr 25;16:20. doi: 10.1186/s13033-022-00530-1 (PMC9036722; doi:10.1186/s13033-022-00530-1)
Supplement: Supplementary file 1 — Additional file 1: Appendix S1. Site visit 1 agenda. [file 13033_2022_530_MOESM1_ESM.pdf]

THE UNIVERSITY OF SYDNEY
Brain and Mind Centre

Right care, first time, where you live

SITE VIST 1 AGENDA

DATE:
TIME:
VENUE:

Attendees:
Brain and Mind Centre:
Site representatives and stakeholders:

No.	Item	Time
1.	**Welcome and Introductions**<br>- Acknowledgement of Country<br>- Acknowledgement of Lived Experience	15 min
2.	***Right care, first time, where you live* Program update**<br>- Program overview and progress<br>• Identification of key individuals to include in workshops and evaluation<br>- Additional offerings of the Program (Digital Technology and BMC Youth Model training)<br><br>- **Site visit schedule**<br>• **Site visit 2 - September: Engagement Team** (participatory modelling process, evaluation, additional Program offerings, economic data preparation)<br>• Need to establish who will be present at visit 2<br>• Are there any key organisations the research team can meet/visit?<br>• **Site visit 3 - October: Technical Team** (systems modelling data preparation)<br><br>- **Annual Symposium (November)**	30 min
3.	**Implementation phase preparation**<br><br>- Time commitment summary<br>- Who needs to be in the room?<br>- Who are the key policy makers?<br>- Who are the key contacts for access to key datsets?<br>- Community engagement – best and preferred approach? Is there a current and preferred team member?<br>- Is there a media contact for the region?<br>- Program check-points<br>• evaluation processes<br>• implementing Digital Technology and BMC Youth Model<br>• which collaborative and local governance mechanism will support this?<br>- Agreements (Research Collaboration Letter and Services Agreement)	40 min
4.	**Round-up discussion and action items**	5 min
