# Supplementary material for: Applying systems approaches to stakeholder and community engagement and knowledge mobilisation in youth mental health system modelling
Source: Int J Ment Health Syst. 2022 Apr 25;16:20. doi: 10.1186/s13033-022-00530-1 (PMC9036722; doi:10.1186/s13033-022-00530-1)
Supplement: Supplementary file 2 — Additional file 2: Appendix S2. Site visit 2 agenda. [file 13033_2022_530_MOESM2_ESM.pdf]

THE UNIVERSITY OF SYDNEY
Brain and Mind Centre

Right care, first time, where you live

SITE VISIT 2 AGENDA

DATE:
TIME:
VENUE:

Attendees:

Brain and Mind Centre:

Site representatives and stakeholders:

(Ensure youth lived experience is included in these initial conversations)

Chair: Dr Louise Freebairn

No.	Item	Time
**1.**	**Welcome and Introductions**<br>- Acknowledgement of Country<br>- Acknowledgement of Lived Experience	15 min
**2.**	**Implementation phase preparation**<br>- Overview of the implementation process<br>- Time commitment summary<br><br>**Local mental health context discussion** (Delegates to be decided by site)<br>- Mental health challenges and resources for the local community/context<br>- Are there any current commissioning priorities that might either fit into or impact on this Program<br>- Priority questions for modelling<br><br>**Participatory modelling process**<br>- Confirming the stakeholders participating in the participatory modelling process<br>- Proposed roles, expectations through the participatory process<br>- Community engagement champion role<br>- Identification of superusers of systems modelling	90 min
**3.**	**Implementation of additional offerings**<br>Supporting local communities with enhanced models of youth mental health care through sophisticated digital technologies	15 min
**4.**	**Evaluation preparation**<br>- Overview of the evaluation process (purpose & timeline)<br>- Community recruitment approach<br>- Identification of key individuals<br>- Demonstration of online surveys	25 min
**4.**	**Economic data preparation**<br>- Overview of the economic analysis<br>- Delegation of 1 or 2 specific staff to have separate meetings to collate program expenditure and clarify data needs and formats	25min
**5.**	**Round-up discussion and remaining action items**	10 min
