# Supplementary material for: Applying systems approaches to stakeholder and community engagement and knowledge mobilisation in youth mental health system modelling
Source: Int J Ment Health Syst. 2022 Apr 25;16:20. doi: 10.1186/s13033-022-00530-1 (PMC9036722; doi:10.1186/s13033-022-00530-1)
Supplement: Supplementary file 3 — Additional file 3: Appendix S3. Site visit 3 agenda. [file 13033_2022_530_MOESM3_ESM.pdf]

THE UNIVERSITY OF SYDNEY
Brain and Mind Centre

Right care, first time, where you live

SITE VISIT 3 AGENDA

DATE:
TIME:
VENUE:

Attendees:

Brain and Mind Centre:

Site representatives and stakeholders:
(Delegates who are Executive lead and data specialists on site)

Chair: Dr Louise Freebairn

No.	Item	Time
1.	**WELCOME:**<br>- Acknowledgement of Country<br>- Acknowledgement of Lived Experience	10 mins
2.	**Systems modelling preparation**<br>- Early discussion of model scope and decision support priorities<br>- Scoping the model including outcomes and interventions of interest<br>- Review of the identification of diverse stakeholder participants<br>• Confirming stakeholders that will be included in the implementation phase<br>• Identification of systems modelling superusers<br>- Discussing any resource needs and planning required for research team to support<br>• Evaluation update & feedback	50 mins
3.	**Data preparation**<br>- Data preparation and discussion of shell tables<br>- Request for sites to complete shell tables<br>- Shell tables will need to be contextualised based on how model will be stratified per region<br>- Early data needs	60 mins
4.	**Economics**<br>- Identification of datasets<br>- Shell tables	30 mins
4.	**Round-up discussion and action items**	30 mins
